# Supplementary material for: Identification of photoperiod-induced specific miRNAs in the adrenal glands of Sunite sheep (Ovis aries)
Source: Front Vet Sci. 2022 Jul 22;9:888207. doi: 10.3389/fvets.2022.888207 (PMC9354845; doi:10.3389/fvets.2022.888207)
Supplement: Supplementary file 1 [file Data_Sheet_1.ZIP › Supplementary file/miRDeep2 file.zip!/miRDeep2 file/Know_Structure/LP42A3/oar-mir-199a.pdf]

miRBase precursor : oar-mir-199a
Total read count : 110316
oar-miR-199a-3p read count : 67697
remaining reads : 42619

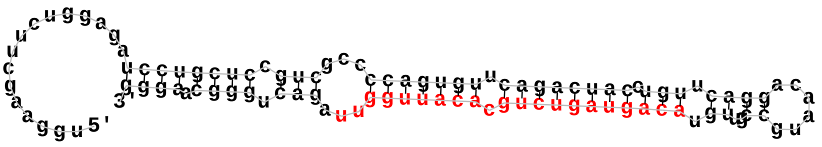

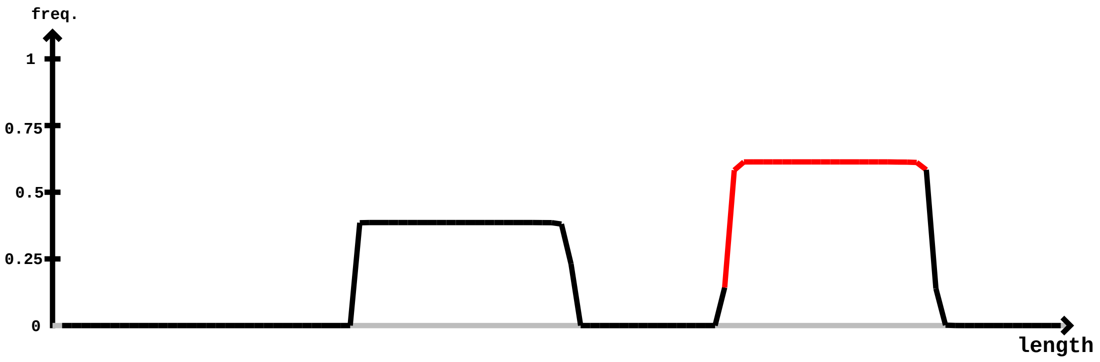


```
                                                                                    oar-miR-199a-3p
5'- uggaagcuucuggagauccugcuccgucgccccaguguucagacuaccuguucaggacaaugccguuguacaguagucugcacauugguuagacugggcaaggg   -3'    exp
    .................(((((((.(((...(((((((.((((((((.(((.((((......))..)).))))))))))).)))))))...))).)))).))).    reads    mm    sample
    .............................ccccaguguucagacuaccuguu....................................................    3        0     seq
    .............................ccccaguguucagacuaccuguuc...................................................    4        0     seq
    ..............................cccaguguucagacuac.........................................................    2        0     seq
    ..............................cccaguguucagacuacc........................................................    2        0     seq
    ..............................cccaguguucagacuaccu.......................................................    32       0     seq
    ..............................cccaguguucagacuaccug......................................................    27       0     seq
    ..............................cccaguguucagacuaccugu.....................................................    583      0     seq
    ..............................cccaguguucagacuaccuguu....................................................    16443    0     seq
    ..............................cccaguguucagacuaccuguuc...................................................    25426    0     seq
    ...............................ccaguguucagacuaccugu.....................................................    2        0     seq
    ...............................ccaguguucagacuaccuguu....................................................    33       0     seq
    ...............................ccaguguucagacuaccuguuc...................................................    55       0     seq
    ................................caguguucagacuaccuguu....................................................    2        0     seq
    ................................caguguucagacuaccuguuc...................................................    4        0     seq
    .....................................................aggacaaugccguuguacaguag............................    1        0     seq
    ...................................................................guacaguagucugcacauugguu..............    1        0     seq
    ....................................................................uacaguagucugcacauu..................    19       0     seq
    ....................................................................uacaguagucugcacauug.................    8        0     seq
    ....................................................................uacaguagucugcacauugg................    70       0     seq
    ....................................................................uacaguagucugcacauuggu...............    1558     0     seq
    ....................................................................uacaguagucugcacauugguu..............    12883    0     seq
    ....................................................................uacaguagucugcacauugguua.............    1196     0     seq
    ....................................................................uacaguagucugcacauugguuag............    9        0     seq
    .....................................................................acagCagucugcacauu..................    1        1     seq
    .....................................................................acaguagucugcacauu..................    41       0     seq
    .....................................................................acaguagucugcacauug.................    11       0     seq
    .....................................................................acaguagucugcacauugg................    76       0     seq
    .....................................................................acaguagucugcacauuggu...............    1363     0     seq
    .....................................................................acaguagucugcacauugguu..............    33940    0     seq
    .....................................................................acaguagucugcacauugguua.............    12922    0     seq
    .....................................................................acaguagucugcacauugguuag............    140      0     seq
    .....................................................................acaguagucugcacauugguuaga...........    16       0     seq
    .....................................................................acaguagucugcacauugguuagG...........    2        1     seq
    ......................................................................caguagucugcacauugg................    7        0     seq
    ......................................................................caguagucugcacauuggu...............    116      0     seq
```

oar-miR-199a-3p

```
uggaagcuucuggagauccugcuccgucgccccaguguucagacuaccuguucaggacaaugccguuguacaguagucugcacauugguuagacugggcaaggg
......................................................................caguagucugcacauugguu..............    2300    0    seq
......................................................................caguagucugcacauugguua.............    987     0    seq
......................................................................caguagucugcacauugguuag............    12      0    seq
.......................................................................aguagucugcacauuggu...............    1       0    seq
.......................................................................aguagucugcacauugguu..............    11      0    seq
.......................................................................aguagucugcacauugguua.............    7       0    seq
```
